# New Genes Causing Hereditary Parkinson’s Disease or Parkinsonism

**DOI:** 10.1007/s11910-017-0780-8

**Published:** 2017-07-21

**Authors:** Andreas Puschmann

**Affiliations:** 1 Lund University, Skåne University Hospital, Department of Clinical Sciences Lund, Neurology, Lund, Sweden; 2 Department for Neurology, Skåne University Hospital, Getingevägen 4, 224 67 Lund, Sweden

**Keywords:** Parkinson’s disease, Parkinsonism, Dominant, Recessive, Monogenic, Genetic

## Abstract

**Purpose of Review:**

This article reviews genes where putative or confirmed pathogenic mutations causing Parkinson’s disease or Parkinsonism have been identified since 2012, and summarizes the clinical and pathological picture of the associated disease subtypes.

**Recent Findings:**

Newly reported genes for dominant Parkinson’s disease are *DNAJC13*, *CHCHD2*, and *TMEM230*. However, the evidence for a disease-causing role is not conclusive, and further genetic and functional studies are warranted. *RIC3* mutations have been reported from one family but not yet encountered in other patients. New genes for autosomal recessive disease include *SYNJ1*, *DNAJC6*, *VPS13C*, and *PTRHD1.* Deletions of a region on chromosome 22 (22q11.2del) are also associated with early-onset PD, but the mode of inheritance and the underlying causative gene remain unclear. *PODXL* mutations were reported in autosomal recessive PD, but their roles remain to be confirmed. Mutations in *RAB39B* cause an X-linked Parkinsonian disorder*.*

**Summary:**

Mutations in the new dominant PD genes have generally been found in medium- to late-onset Parkinson’s disease. Many mutations in the new recessive and X-chromosomal genes cause severe atypical juvenile Parkinsonism, but less devastating mutations in these genes may cause PD.

## Introduction

Since the discovery of the first gene for Parkinson’s disease (PD) 20 years ago, a large number of additional genes have been implicated as monogenic causes for PD, or for disorders with Parkinsonism as a more or less prominent clinical feature. Today, genetic testing of patients with young-onset, hereditary or unusual Parkinsonian disorders has become part of clinical practice in many healthcare settings. This review attempts to evaluate the reports since 2012 on new genes with mutations associated with monogenic PD or Parkinsonism, concentrating on the clinical and pathological descriptions of these new forms of disease. For reasons of space, this review does not include new results on PD genes identified before 2012, where new results acquired in recent years have consolidated, expanded, and sometimes refuted previous knowledge. Readers are referred to broader previous reviews that summarize the knowledge in the field available at the time of their writing [1–6].

## New Genes for Dominant PD

Since 2012, mutations in *DNAJC13*, *CHCHD2*, *TMEM230*, and *RIC3* have been reported as new causes for monogenic dominant PD. The following sections provide a review of the available reports on the clinical picture and genetic information. All of these discoveries are recent, and it is today not definitely proven that mutations in these genes cause PD. The present evidence appears most robust for a causative role of *CHCHD2* mutations in PD because of the description of more than one family with co-segregation of clinical phenotype and genotype, but less robust for *DNAJC13* and *TMEM230*, because these mutations were found in only one, albeit large, pedigree, and least robust for *RIC3* that has only been reported from one less extensive family. Table 1 provides an overview over presently known genes for dominant PD, which generally cause medium- to late-onset Parkinsonism or PD, for most genes with few or no additional features.

**Table 1:** Monogenic causes for autosomal dominant Parkinson’s disease

Gene	Year	Additional clinical features besides Parkinsonism
***SNCA***	1997	Cognitive, behavioral, and autonomic dysfunction, myoclonus
***LRRK2***	2004	Few, variable. Possibly only milder non-motor disturbances
*EIF4G1*	2011	Probably not pathogenic
***VPS35***	2011	Cognitive and behavioral changes (?)
*DNAJC13*	2013	None
*CHCHD2*	2015	None. Two carriers with tremor only
*TMEM230*	2016	One large family only
*RIC3*	2016	One family only

Gene names not in bold print indicate that pathogenicity remains unconfirmed

### DNAJC13

In 2013, Vilariño-Güell et al. identified c.2564A>G (p.N855S) mutations in *DNAJC13* in a large family with autosomal dominant PD. Eleven PD patients from two successive generations carried these mutations, whereas one family member with progressive supranuclear palsy and two additional members with PD did not. DNAJC13 p.N855S mutations were also identified in two additional familial and three sporadic cases from Canada [7••, 8], but not in other populations so far [9–11]. A recent follow-up study identified three additional members of the original family who had become symptomatic by that time [12]. All known carriers reside in Saskatchewan or British Columbia, Canada, and reported Dutch-German-Russian Mennonite origin, strongly suggesting a common founder. This was supported by results of genetic comparisons of the haplotype surrounding the disease-causing mutation [7••].

The clinical phenotype of DNAJC p.N855S carriers was described as clinically definite Parkinson’s disease in 13 out of the 17 patients reported so far [8, 12]. The remaining four patients had relatively mild disease with rigidity and bradykinesia as well as action or postural tremor, but no resting tremor. Six patients, including all who only had action or postural tremor, did not require levodopa treatment at all, despite disease durations of up to 20 years. Symptom onset was between 40 and 85 years (mean 63.2; standard deviation, S.D., 12.5), and disease progression was generally slow. Patients in earlier disease stages responded to levodopa, but patients at late disease stage (Hoehn and Yahr 4) did not [12]. Multitracer PET examinations revealed a dopaminergic deficit in the striatum with a pattern as in idiopathic PD, affecting the putamen more than the caudate [12]. Three mutation carriers from the original kindred have been examined neuropathologically after disease durations of 8 to 17 years. All had alpha-synuclein-positive Lewy body pathology with cell loss in the basal nucleus of Meynert and substantia nigra, and the distribution of alpha-synuclein pathology was brainstem only in one and transitional in two. All three showed tau pathology with neurofibrillary tangles including hippocampal involvement and arteriosclerotic vascular disease. Two patients had evidence of cerebrovascular accidents [7••, 12]. DNAJC13 p.N855S mutations are rare events [10].

Another mutation in *DNAJC13*, c.6344G>T (p.R2115L), was identified in two patients and one unaffected member of one Tunisian family [7••]. It is uncertain if this mutation is pathogenic. DNAJC13 protein is present on endosomal membranes where it is involved in clathrin coating of early endosomes. p.A855S-mutated DNAJC13 impairs endosomal transport by a toxic gain-of-function mechanism [7••].

No coding variants in *DNAJC13* were detected when the gene was sequenced in 1938 Caucasian PD cases and 838 patients with pathologically confirmed Lewy body disease [10]. Variants in the gene have been identified as PD susceptibility factors [8, 11].

### CHCHD2

In 2015, Funayama et al. reported *CHCHD2* c.182C>T (p.T61I) mutations in a large Japanese family with ten affected mutation carriers in two successive generations. The same mutation was found in a different family with autosomal dominant PD where, interestingly, one of the mutation carriers had fine tremor since age 10, but no Parkinsonism at age 50 [13••]. Seven of nine mutation carriers with PD had been treated with levodopa, and all had good response. Two of ten patients described had hyperreflexia. This mutation was also found in two affected siblings from a Chinese family with seemingly autosomal dominant PD. Also in this family, a third sibling carried the p.T61I mutation and had only mild tremor from age 39 and a symptomatology compatible with essential tremor 3 years later, without overt Parkinsonism [14]. PET studies showed mildly reduced dopamine reuptake in the posterior putamen in this individual, and more markedly reduced dopamine uptake in the putamen and the caudate nucleus in an affected brother [15]. Two of seven patients from the Chinese family had dysphagia, one displayed a positive Babinski sign and one had electrophysiological evidence for neurogenic muscle degeneration. The average age at symptom onset in the three families combined was 52 years (S.D. 5.8; range 40–61) [13••, 14], and typically there was asymmetric bradykinesia, rigidity, and resting tremor that responded well to levodopa. No neuropathology of patients with *CHCHD2* mutations has so far been reported.

Several other studies have since examined the *CHCHD2* gene in patients and controls. From Germany, a PD patient with *CHCHD2* c.376C>T (p.Gln126X) mutation has been reported and this mutation has been suggested to be pathogenic as it leads to protein truncation [16]. Other rare variants mutations in *CHCHD2* have been identified in PD patients, but their pathogenicity remains more uncertain [13••, 16, 17], and *CHCHD2* variants have been shown to increase PD risk [18]. In total, more than 4100 PD cases and more than 1900 unaffected controls have been analyzed to date [13••, 14, 16–25]. Definite confirmation of the pathogenicity of *CHCHD2* mutations is currently lacking.

### RIC3

In a three-generational family from Karnataka State in Southern India, Sudhaman et al. identified RIC3 c.169C>A (p.P57T) mutations [26]. In this family, five members in two generations had bradykinesia and rigidity, and two of these also tremors and the oldest individual had Parkinsonism and dystonia. The average age at onset was 60 years (S.D. 5.2, range 54–68). Four of these individuals also had restless legs syndrome, and two REM-sleep behavior disorder. An additional member with PD was deceased at the time of study. Four members of the successive generation displayed very mild signs such as reduced arm swing, bradykinesia, and/or rigidity on clinical examination at age 26 to 40 years, while five of their siblings and cousins did not show any movement disorder. RIC3 p.P57T mutations were present in all nine affected members and absent in all five unaffected members tested [26]. The fact that only mild symptoms were reported from the only reported family’s third generation opens up for the possibility that some of these individuals may not develop PD, and conversely, some of their younger siblings or cousins may still develop the disease. No additional kindred with mutations in *RIC3* has so far been identified; why the pathogenicity of *RIC3* mutations for PD remains unconfirmed. Variants in *RIC3* have not been associated with PD in one case-control study performed to date [27].

### TMEM230

In 2016, Deng et al. reported the results of their independent analysis of the same Canadian Mennonite kindred where Vilariño-Güell et al. previously found mutations in *DNAJC13* (see above) [7••, 28••]*.* Deng et al. instead identified *TMEM230* c.422G>T (p.R141L) as the cause of the disease in that kindred. They emphasized the fact that two family members with PD and one with progressive supranuclear palsy did not carry *DNAJC13* mutations, whereas all affected individuals they tested carried *TMEM230* mutations. No additional large family with clear co-segregation of a *TMEM230* mutation and PD has been identified.

Deng et al. also identified three additional *TMEM230* variants in PD patients, among them *TMEM230* c.550_552delTAGinsCCCGGG (p.*184ProGlyext*5). This mutation was unusually common among Chinese PD families, reported from 7 out of 574 families, who were either homozygous or heterozygous carriers, and co-segregated with PD in two affected sib-pairs from these seven families [28••, 29]. Several additional studies examining large series of PD patients, mostly of Caucasian and Chinese origin, have remained negative and have not identified the p.*184ProGlyext*5 variant [30–39]. The pathogenicity of *TMEM230* variants needs to be considered unconfirmed.

## New Genes for Recessive and X-Linked PD or Parkinsonism

Compared to monogenic dominant PD and to the well-established recessive early-onset PD genes *PARK2*, *DJ-1*, and *PINK1*, the newly identified recessive forms appear more complex both for clinicians and researchers. The clinical picture of the newly identified recessive forms is often more severe and multifaceted. However, in a few instances, there appears to be a genotype-phenotype correlation where mutations that lead to pronounced alteration of normal protein function cause a complex disorder with severe additional neurological or neuropsychiatric impairment, often from birth, and juvenile Parkinsonism, whereas mutations with a milder effect on the protein cause Parkinsonism with fewer atypical features. Table 2 summarizes currently known and putative genes for recessive and X-linked PD or Parkinsonism.

**Table 2:** Monogenic causes for autosomal recessive or X-linked Parkinson’s disease or atypical juvenile Parkinsonism

Gene	Year	Atypical clinical features
***PARK2*** **(** ***Parkin*** **)**	1998	None
***DJ-1***	2003	None. Psychiatric features, dystonia (in some patients)
***PINK1***	2004	None. Cognitive/psychiatric symptoms (in some patients)
***FBXO7***	2008	Spasticity, equinovarus deformity (in some patients)
***22q11.2del***	2013	Intellectual disability
***SYNJ1***	2013	Dystonia, cognitive decline, seizures
***RAB39B***	2014	Severe intellectual disability. X-linked mode of inheritance
***DNAJC6***	2015	Intellectual disability, other neurological features
*PODXL*	2016	None. One family only. To be confirmed in independent studies
***VPS13C***	2016	Severe cognitive dysfunction, tetraplegia
***PTRHD1***	2017	Severe cognitive dysfunction, muscular weakness

Mutations in additional genes have long been known to cause recessive neurological diseases that in various proportions of patients may cause Parkinsonism [2, 4]. These syndromes are not included here since they are clearly distinguishable from PD clinically, and/or not newly identified since 2012. Gene names not in bold print indicate that pathogenicity remains unconfirmed

### 22q11.2 Deletion Syndrome

Hemizygous deletions of a segment within the 11.2 band on the long arm of chromosome 22 are known to cause a clinical syndrome, 22q11.2 deletion syndrome, with very variable signs and symptoms, often including learning difficulties, midline developmental abnormalities, especially of the palate, larynx, trachea, and/or esophagus, subtle facial dysmorphies, and deficiencies of the immune system [40]. Autism spectrum disorder or schizophrenia is a common manifestation, as well as relatively milder disturbances such as difficulties with social interaction, anxiety, or attention deficit disorder. There may be hypocalcemia and renal or skeletal abnormalities. The designation 22q11.2 deletion syndrome includes phenotypes previously known as velocardiofacial syndrome and DiGeorge syndrome. 22q11.2 deletion syndrome is considered to be the most common microdeletion syndrome in humans, occurring in more than 1 per 4000 births. The majority of patients have a deletion of 3 million base pairs, encompassing about 40 genes, but a minority have shorter nested deletions. Many of the anomalies are present from birth, but some of the other symptoms, especially the psychiatric symptoms, may develop in mid-adult life [40].

Parkinsonism had already been described between 1998 and 2010 in four patients with this syndrome [41–43]. In 2013, Butcher et al. reported additional three unrelated patients with 22q11.2 deletion syndrome and Parkinsonism, and, importantly, provided the results of a neuropathological examination of three patients [44]. Two of these had cell loss in the substantia nigra and prominent alpha-synuclein-positive Lewy bodies in the brainstem; one patient had marked cell loss in the substantia nigra, locus coeruleus, and dorsal nucleus of the vagal nerve but without alpha-synuclein pathology. There was also amyloid and tau pathology. The findings confirmed the presence of the neurodegenerative process typical for PD and ruled out that all Parkinsonian signs and symptoms were solely induced by neuroleptic treatment for the psychiatric disease manifestations. Case reports and small case series on six additional patients have been published since [45–49].

The clinical phenotype of these patients included Parkinsonism, starting at a mean of 41 years (S.D. 8) of age, often with resting tremor or gait disturbance. There was good response to levodopa or other dopaminergic treatment, which often was combined with withdrawal of antipsychotic medication, and early development of motor fluctuations was reported. Frequently, worsening of psychotic symptoms limited the dose of dopaminergic medications. However, all patients reported in these reports had marked additional symptoms such as severe and difficult-to-treat psychosis or schizophrenia, learning disabilities, or obvious midline imaging anomalies. These additional features were usually present from birth or childhood or young adult age, long before Parkinsonian signs were noted. SPECT examinations have been reported from six patients who all showed bilaterally reduced dopaminergic reuptake in the striatum [43, 48, 50]. MRI brain imaging has shown cava septi vergae, white matter hyperintensities in T2, and reduced brainstem volume in these case reports. MIBG scintigraphy was performed in one patient and showed normal cardiac autonomous innervation [48].

In a large multicenter case-control study, Mok et al. addressed the question whether hemizygous 22q11.2 deletions are associated with idiopathic PD. Among 9387 patients enrolled in their case series with a clinical diagnosis of PD, eight were found to be hemizygous carriers of 22q11.2 deletions; no such deletion was found among 13,863 control subjects [50]. The eight mutation carriers had developed Parkinsonian signs or symptoms at a median of 37 years. Given the large scale of the study, detailed individual data was not available for each of the mutation carriers, but retrospectively other features of 22q11.2 deletion syndrome were present in all of the mutation carriers, although they may have been mild at the time of inclusion in the study, and may not have been specifically asked for at the time of enrollment in research studies focused on PD [50].

Patients with hemizygous 22q11.2 deletions also showed additional movement disorder phenomena, such as postural and action tremor, myoclonic jerks, and intermittent oculogyric movements, alone or in combination with Parkinsonian signs [46]. Medical treatment of the symptoms of 22q11.2 deletion syndrome requires a careful balance of antiparkinsonian and antipsychotic agents, and the readiness to accept suboptimal treatment responses regarding both Parkinsonism and psychosis.

Presently, it is not known which mutation(s) in which of the genes in the deleted segment causes the development of Parkinsonism in these patients, or if the microdeletion per se is causative. Several possible candidate genes have been suggested [51]. All patients with 22q11.2 deletion syndrome for whom the extent of the deletion was examined showed forms of the long, 2.8 to 3.0 million base pair deletion, and not the rarer shorter deletions [45, 47, 50]. One additional PD patient with a shorter, atypical deletion of only 726 kb length was reported but not considered to represent 22q11.2 deletion syndrome; this deletion overlapped only to a small part with the area usually deleted in the shorter deletions [30].

Investigation for 22q11.2 deletions was suggested in the diagnostic workup of patients with early-onset PD who have congenital palatal or cardiac defects, recurrent infections or other signs of immune deficiency, late developmental milestones, or marked psychiatric comorbidity [49]. It has been pointed out that early detection of 22q11.2 deletions might be beneficial for timely recognition and treatment of manifestations such as hypocalcemia, abnormal thyroid or parathyroid function, abnormal magnesium levels, or cardiac abnormalities [49].

### SYNJ1

Quadri et al. and Krebs et al. simultaneously described two families with homozygous mutations c.773G>A (p.R258G) in the *SYNJ1* gene [52••, 53••]. In each of the two families, one from Sicily and one from Iran, there were two affected siblings, and parents were consanguineous. In the Italian family, one son developed slowness of movements, leg stiffness, fatigue, and upper arm involuntary movements at age 22 years. He soon became unable to walk and to work and within 3 years lost his ability to talk. Dystonia, irregular tremor, eyelid apraxia, and supranuclear vertical gaze limitation were noted. Levodopa was ineffective but worsened the oromandibular and limb dystonia. The patient had severe cognitive dysfunction. Symptom progression was unusually rapid initially, but after more than two decades, the disorder remained generally stable, with some degree of fluctuation [54]. His sister had similar but perhaps somewhat milder symptoms starting at 28 years. MRI showed cerebral cortical atrophy, quadrigeminal plate thinning, and hippocampal T2 hyperintensity. Further imaging showed nigrostriatal dopaminergic deficit and cortical hypometabolism [52••]. The two siblings from the Iranian family additionally had seizures during infancy and from the age of 3 years, respectively. The developed a severe progressive Parkinsonian syndrome with chin tremor in their 20s. They had oculomotor abnormalities and severe hypophonia. A short but marked improvement after administration of low levodopa doses (25 mg) was noted in one of the patients whereas his sister developed dyskinesia at the same dose. Cognitive function was considered normal. MRI showed cortical atrophy and white matter abnormalities. One of the siblings had a meningioma, impressing on the brainstem why interpretation of the symptoms is difficult [53••].

A third family with the same SYNJ1 R258G mutation, also from Southern Italy, was identified soon thereafter [55]. Their clinical presentation was similar to the previous families, but the two siblings had reached developmental milestones of childhood development somewhat late. No abnormalities on MRI could be discerned. Follow-up examinations after 4 and 7 years of disease duration showed progress of Parkinsonism, albeit not unusually rapid, depression, and progressive cognitive impairment. There were no indications for REM-sleep behavioral disorder, and MIBG scintigraphy showed normal cardiac sympathetic innervation [56].

A different *SYNJ1* mutation, c.1376C>G (p.R459P), has since been identified in a consanguineous Indian family from Jharkhand state [57]. The two affected individuals had developed tremor and at age 12 and 18 years developed Parkinsonism that was alleviated by levodopa, dystonia, and dyskinesia but had not developed dementia after 4 and 20 years of disease duration.

Two German siblings, compound heterozygous for two different *SYNJ1* mutations c.512G>A (p.W171*) and c.773G>A (p.R258G), had seizures within the first 4 years of life, and developed generalized, dopa-responsive dystonia at ages 13 and 15 years, with action tremor of the tongue, head, and extremities. One of them had reached developmental milestones late, both had cognitive dysfunction in early adult life. CSF analyses showed signs associated with impairments in dopamine synthesis, including decreased homovanillic acid and tetrahydrobiopterin [58].

Other *SYNJ1* mutations cause a recessive epilepsy syndrome with progressive and very severe neurological decline during the first years of life [59, 60]. It has been noted that mutations associated with Parkinsonism affect the protein’s SAC1-like domain, whereas mutations causing the severe epilepsy syndrome impair the protein’s dual phosphatase activity [53••, 55, 57–60].

No pathology has been reported from a patient with *SYNJ*-related Parkinsonism, but one Pakistani child with SYNJ1 c.406C>T, p.R136X mutations, and a most severe neurodevelopmental syndrome with intractable epilepsy was examined neuropathologically after death at age 6.5 years. Macroscopically, there was general white matter atrophy but, remarkably, the most prominent pathology on microscopic examination was found in the substantia nigra: There was marked cell loss, and intensely positive tau pathology with neurofibrillary tangles, in cell bodies, axonal hillocks, and neuropil threads. Similar but milder pathology was found in the basal ganglia. There was no immunoreactivity to alpha-synuclein [59]. The parkinsonian patients with *SYNJ1* mutation showed clinical and radiological signs reminiscent of other tauopathies, such as vertical gaze palsy and atrophy of the quadrigeminal plate, suggesting that a tauopathy might be the underlying cause.

Recently, Taghavi et al. reported a SYNJ1 p.R839C mutation in a small Iranian family with two affected siblings with poorly levodopa-responsive Parkinsonism and generalized seizures since 24 years of age. Longitudinal fissures were present on the tongue of this family’s index patient and were thereafter also noticed on the tongue of a previously described Iranian patient carrying SYNJ1 p.R258G [53••, 61], why the authors suggest this might be an additional disease sign.

### RAB39B

In 2014, Wilson et al. reported deletions of the entire *RAB39B* gene on the X chromosome in an Australian family with three affected brothers [62••]. These individuals had intellectual disability from birth with delayed speech initiation and early learning difficulties in all three, to a degree that independent living was impossible. One individual also had delayed early motor milestones as well as obsessional and ritualistic behavior [62••]. Tremor was noted from late childhood in one of the three brothers, and the other two had tremor by age 38 and 44 subsequently developed akinetic-rigid Parkinsonism which was responsive to levodopa. One of these patients with Parkinsonism had side effects to treatment, and mild T2 MRI signal changes in the substantia nigra and globus pallidum that were interpreted as deposition of iron or calcium. This individual came to autopsy at age 48, which revealed cell loss and alpha-synuclein-positive Lewy bodies in the substantia nigra as well as an abundance of cortical alpha-synuclein-positive Lewy bodies. Also, there were some tau-positive neurofibrillary tangles in the substantia nigra, and rare axonal spheroids in the white matter tracts of the basal ganglia area [62••]. The pathology of this patient was thus compatible with the pathology that typically defines PD [63], and added *RAB39B*-related disease to the list of synucleinopathies [64].

Wilson et al. also found c.503C>A (p.T168K) mutations in the *RAB39B* gene in a large family from Wisconsin, USA, where seven male members from one generation were affected. The Wisconsin family was originally described in 1985, and all affected members had intellectual disability, persistent frontal lobe reflexes, tremor, rigidity, and Parkinsonian postural abnormalities [65]. Seizures, strabismus, and macrocephaly occurred in some members. By linkage analysis, the genetic locus of the disorder in the Wisconsin family had been narrowed down to the distal long arm of the X chromosome [66], where *RAB39B* is located.

Mutations in *RAB39B* had already in 2010 been identified in (other) families with X-linked intellectual disability associated with autism, epilepsy, and macrocephaly: In one large kindred (designated MRX72) from Sardinia, Italy, eight male members displayed moderate to severe intellectual disability and global delay of all psychomotor development, three of whom had seizures and one an autism spectrum disorder [67]. Affected members had previously been reported to carry an intronic mutation, c.215+1G>A, in a 5′ splice site of *RAB39B* that affects normal splicing and disrupts protein biosynthesis [68]. A second family (named Fam. X, or D-23) carried *RAB39B* c.21C>A (p.Y7X) mutations that introduce an early stop codon [68]; six members in two generation had mild to severe intellectual disability and macrocephaly, two of which had autism, and four had short stature [68]. Interestingly, duplications and triplications of *RAB39B* lead to intellectual disability and behavioral disturbances; these mutations have been described in children, some of whom also had mild motor symptoms or reached milestones of motor development late [69, 70]. However, no parkinsonian features were reported from any of these families.

Additional individuals and families with *RAB39B* and Parkinsonism have been described: *RAB39B* c.574G>A (p.G192R) mutations co-segregated with disease in a large US family of European origin [71], compatible with X-linked dominant inheritance. Seven affected members had Parkinsonism with symptoms developing between 29 and 53 years in five males and at 55 and 57 in two females. Heterozygous mutations were found in females who were clinically unaffected, seen as evidence for incomplete penetrance and later disease onset in female compared to male mutation carriers. Mild lifelong intellectual disability was documented for two of the affected members, but not for the other; however, the article refrains from describing the methods for clinical assessment, and mostly typical PD motor manifestations are described. Based on the published information, it cannot be excluded with certainty that more detailed investigations for non-motor signs, or psychological assessment, would have revealed intellectual disabilities in these patients as well.

*RAB39B* c.536dupA (p.E179fsX48) was identified in a small Chinese family with two male patients who had learning difficulty from childhood and mild intellectual disability [72]. Both developed tremor and rigidity at age 10 and 12 years, progressing to moderate or severe PD by ages 20 and 58 years. Levodopa treatment had a mild effect on the younger patient but not the older one. The older individual had severe cognitive decline at age 58. Both patients showed calcifications of the globus pallidus in CT.

*RAB39B* mutations have also been found in additional single individuals [71, 73]. Detailed clinical information is provided on one French patient with *RAB39B* c.557G>A (p.W186X) who had mild intellectual disability, requiring sheltered employment, and who developed asymmetric tremor at rest and akinetic-rigid Parkinsonism at age 39 years. By age 47, he had developed motor fluctuations, dyskinesia, and dystonia, and dysthymia with impulsiveness to a degree that he was considered unsuitable for DBS surgery [73].

Thus, *RAB39B* mutations are a well-established cause for intellectual disability, and Parkinsonism, with alpha-synuclein-positive pathology, was observed in some of these patients.

The RAB39B mutations described in patients who developed Parkinsonism are loss-of-function mutations. RAB39B belongs to the Rab GTPase superfamily of proteins that regulate vesicular trafficking. RAB39B is present in high amounts in neurons where it also appears to be involved in the vesicular recycling pathways between the synaptic cell membrane, endosomes, and the trans-Golgi network. Furthermore, RAB39B may have a role in maintaining intraneuronal alpha-synuclein homeostasis [62••, 68, 71].

### DNAJC6

In 2012, Edvardson et al. described a homozygous intronic c.801-2A>G mutation in the *DNAJC6* gene that affects normal splicing, in two affected siblings of a Palestinian family [74•]. The two patients had normal early development, but signs of childhood-onset Parkinsonism were noted at the age of 7 and 11 years. Both patients soon developed severe and debilitating disease with pronounced bradykinesia, rigidity, postural instability, and rest tremor, and both were unable to walk at age 13 and 18 years. Pharmacological treatment attempts were made with several types of dopaminergic medication but did not improve symptoms. Hypometric saccades were noted in one of the patients.

Subsequently, the truncating mutation c.2200C>T (p.Q734X) was found in four affected members of a consanguineous Turkish kindred [75]. These individuals had a severe disease with mild to moderate intellectual disability, seizures (in three individuals), Parkinsonism, and pyramidal signs. Age at onset was 10 to 11 years, and all four patients became wheelchair bound or bedridden in their 20s. Motor symptoms included resting and postural tremor, bradykinesia, rigidity, and intermittent dystonia. Levodopa (62.5 mg) had good effect on these symptoms but caused severe motor and psychiatric side effects. A patient who underwent MRI scanning in his 40s had diffuse brain atrophy. Two patients each had scoliosis and myoclonus [75].

By sequencing the *DNAJC6* gene in patients with early-onset PD, Olgiati et al. discovered three patients with double mutations in this gene [76••]. Two of them had familial disease, and analysis of affected and unaffected family members showed complete co-segregation. Moreover, homozygosity analysis and whole-exome sequencing supported the contention that the *DNAJC6* mutations were disease-causing. One Dutch family carried *DNAJC6* c.2779A>G (p.R927G) mutations, and one Brazilian family the c.2223A>T mutation that is not predicted to cause any amino acid change, but there was good evidence that it may affect splicing. Patients developed hand tremor and/or motor slowness at ages between 21 and 42 years. Bradykinesia, rigidity, and postural instability ensued in all four, and rest tremor in three of them. The disease progressed, motor fluctuations developed, and the oldest patient was bound to a wheelchair at 48 years of age. All had good effect of levodopa, but three of the patients developed levodopa-induced hallucinations. In contrast to most of the previously described patients with *DNAJC6* mutations, no intellectual disability was noted. However, one patient required neuroleptic treatment at age 21 because of psychosis, and Parkinsonism developed at this age as a side effect [76••].

In one patient of Sudanese family with Yemeni origin, a homozygous *DNAJC6* c.2365C>T (p.Q789*) was found; there were no additional affected family members, but the four unaffected members examined genetically did not share the same genotype [77]. The patient started to have terrifying visual hallucinations at age 10.5 years, followed by rapidly deteriorating cognition and motor function. Severe rigidity and resting tremor developed, as well as pyramidal signs, and the patient started to have epileptic seizures, and additional psychotic features. Motor function also deteriorated rapidly; at age 12.5, the patient was almost entirely akinetic.

*DNAJC6* is thus a well-established cause of Parkinsonism with childhood onset, often associated with cognitive or psychiatric symptoms. However, a genotype-phenotype effect has been observed where mutations that have a large impact on protein function lead to very severe, early-onset disease, and mutations that affect protein function more mildly cause adult early-onset PD with few, or no, additional signs and symptoms [76••, 78].

### VPS13C

Also in 2016, Lesage et al. reported three apparently sporadic patients with mutations in *VPS13C* [79••]. The first mutation, homozygous c.8445+2T>G, was identified in a Turkish patient by homozygosity linkage mapping and exome sequencing of consanguineous families with early-onset Parkinsonism. Two additional patients from France were compound heterozygous for mutations in *VPS13C*, c.806_807insCAGA/c.9568G>T (p.R269Sfs*14/p.E3190*), and c.4165G>C/c.4777delC (p.G1389R/p.Q1593Kfs*7). The patients all had a relatively similar clinical presentation with an asymmetric akinetic rigid syndrome starting at 25, 33, and before 46 years of age. There was initial or partial effect of levodopa treatment, and two of the patients developed motor fluctuations or dyskinesias. However, progression was rapid and severe. All three patients developed severe and early cognitive dysfunction; two each had dysautonomia or limb dystonia. All patients were bedridden at 31, 43, and 58 years of age, and two developed dysphagia. All three had pyramidal signs or symptoms with hyperreflexia, limb atrophy, or spastic tetraplegia. MRI of the homozygous patients showed asymmetric cerebral atrophy. Neuropathology of one of the patients who died after a 16-year disease duration revealed mild frontal atrophy, and widespread and abundant alpha-synuclein-positive Lewy bodies and neurites, resembling diffuse Lewy body disease. In addition, there was widespread tau-pathology with neurofibrillary tangles. The mutations have been shown to alter mitochondrial function and PINK1/parkin-dependent mitophagy, and to upregulate the response of parkin to mitochondrial damage [79••].

### PODXL

In an Indian family with three affected siblings from the state of Uttar Pradesh, Sudhaman et al. recently identified a homozygous frameshift *PODXL* c.89_90insGTCGCCCC that results in complete loss of PODXL protein [80]. Patients developed levodopa-responsive Parkinsonism at 13–17 years of age, developed dyskinesia and off-dystonia, and showed no obvious additional signs. Five additional unaffected siblings as well as the unaffected, consanguineous parents were either heterozygous or did not carry the mutation, which was shown to influence neuronal branching. Confirmation of these findings in independent patients and families is awaited.

### PTRHD1

Two independent reports from Iran have linked *PTRHD1* (*C2ORF79*) to recessive Parkinsonism. Khodadadi et al. detected homozygous *PTRHD1* c.157C>T (p.H53Y) mutations in two siblings with intellectual disability and motor abnormalities from childhood, who developed muscle stiffness, resting and postural tremor, and anxiety, hypersomnia, and hypersexuality [81••]. Parkinsonism developed as a relatively late part of that syndrome in the late 20s to 30s and was improved by levodopa in daily doses of 300 to 450 mg. Jaberi et al. described a second family with two brothers who had a similar clinical presentation [82]. The patients had developed gait disturbance, slowness of movements, tremor, and falls in the mid-20s. Neurological examination yielded parkinsonian signs but also distal muscular atrophy and weakness, hyperreflexia and positive Babinski sign, and evidence for axonal sensorimotor polyneuropathy. One of the patients had severe intellectual disability, the other one attention deficits, difficulty concentrations, and perseveration of speech. Genetic analyses of the second family identified two possible disease causes: one was a mutation in *ADORA1*, and the other one c.155G>A (p.C52Y) in *PTRHD1*. Jaberi et al. attributed the disorder to *ADORA1*, but Khodadadi et al. subsequently considered the mutations in *PTRHD1*, affecting the same protein domain and present in both families with similar clinical pictures, as the true cause of the disease also in the family described by Jaberi et al. Microdeletions of the locus including the *PTRHD1* gene are known causes of syndromes with intellectual disability [81••]. Further confirmation in additional families is desirable.

## Conclusion

A large body of work has nominated several new genes for dominant, recessive, and X-linked PD or Parkinsonism during recent years, and high-throughput technologies such as exome sequencing will continue to suggest new genetic causes in the near future. Some of the genes have been connected to PD or Parkinsonism by a solid body of evidence with observations of the mutation’s co-segregation with clinically similar disease subtype in more than one family. Other, more recently identified or rarer mutations await confirmation. There have been instances where initially suggested genes turned out to be—most probably—not pathogenic after subsequent studies, including the diverging observations on *EIF4G1* mutations in PD patients and healthy subjects [4, 5], as well as the initial identification of *ADORA1* as disease-causing mutations [82] in a family who also have *PTRHD1* mutations, and there is ongoing uncertainty on the truly pathogenic variant in the large Canadian Mennonite kindred discussed above, where *DNAJC13* and *TMEM230* mutations have been postulated to be disease-causing [7••, 28••]. Clinicians need to be aware of possible uncertainties in our present knowledge on these newly discovered genetic entities when providing genetic counseling to patients and families with monogenic forms of PD or Parkinsonism.

Several of the genes discussed above do not cause PD but syndromes with Parkinsonism and other characteristic signs. Despite the addition of these newly identified genes to our lists of PD genes, the majority of cases of familial aggregation of PD remain unexplained, and the search for a monogenic cause for PD in many such families has not been fruitful despite large effort [83]. Alternative explanations may include digenic or oligogenic inheritance, where two or a few genetic variants have a pathogenic effect when they occur together in individuals and some of their relatives [84, 85••]. Such variants need not be extremely rare, and may have an intermediate effect size, so that they by themselves do not cause disease, but will be pathogenic in combination with other genetic variants. They may be in newly discovered genes or in genes that also harbor disease-causing mutations, such as the PINK1 p.G411S variants for whom we and others recently have shown that they markedly increase PD risk, but are not per se disease-causing in all carriers [86–88]. One example of an interaction between two genetic variants has recently been identified, whereby genetic variation in *DNM3* modifies the effect of the common LRRK2 p.G2019S mutations [89]. Research networks with exome or whole-genome data from a large number of patients can be predicted to identify many additional similar variants and complex gene-gene interactions, gradually filling the gap of “missing heritability” in idiopathic PD.
